# LAP2α orchestrates alternative lengthening of telomeres suppression through telomeric heterochromatin regulation with HDAC1: unveiling a potential therapeutic target

**DOI:** 10.1038/s41419-024-07116-4

**Published:** 2024-10-19

**Authors:** Bing Wang, Haomeng Kou, Yuwen Wang, Qi Zhang, Duo Jiang, Juan Wang, Ziqin Zhao, Yao Zhou, Miaomiao Zhang, Lei Sui, Mingfeng Zhao, Yancheng Liu, Yang Liu, Lei Shi, Feng Wang

**Affiliations:** 1 The Province and Ministry Co-sponsored Collaborative Innovation Center for Medical Epigenetics, Department of Genetics, School of Basic Medical Science, Institute of Prosthodontics School and Hospital of Stomatology, General Hospital, Tianjin Medical University, 300070 Tianjin, P. R. China; 2 Department of Hematology, Tianjin First Central Hospital, 300192 Tianjin, P. R. China; 3 Department of Clinical Laboratory, First Affiliated Hospital of Xi’an Jiaotong University, 710061 Xi’an, Shaanxi P. R. China; 4 Department of Pathology, Tianjin Hospital, 300221 Tianjin, P. R. China; 5 Department of Bioinformatics, The Province and Ministry Co-Sponsored Collaborative Innovation Center for Medical Epigenetics, School of Basic Medical Sciences, Tianjin Medical University, 300070 Tianjin, P. R. China; 6 Department of Pathology, Jining No.1 People’s Hospital, 272000 Jining, Shandong P. R. China; 7 Department of Prosthodontics, School and Hospital of Stomatology, Tianjin Medical University, 300070 Tianjin, P. R. China; 8 Department of Bone and Soft Tissue Oncology, Tianjin Hospital, 300221 Tianjin, P. R. China; 9 Department of Radiobiology, Institute of Radiation Medicine, Chinese Academy of Medical Sciences & Peking Union Medical College, 300192 Tianjin, P. R. China; 10 Department of Biochemistry and Molecular Biology, School of Basic Medical Sciences, Tianjin Medical University, 300070 Tianjin, P. R. China

**Keywords:** Telomeres, Bone cancer

## Abstract

In response to the challenge of telomere attrition during DNA replication, cancer cells predominantly employ telomerase or, in 10–15% of cases, the alternative lengthening of telomeres (ALT). The intricate details of ALT, however, remain elusive. In this study, we unveil that the knockdown of lamina-associated polypeptide 2 alpha (LAP2α) in ALT cells results in telomere dysfunction, triggering a notable increase in ALT-associated hallmarks, including high frequencies of PML bodies (APBs), C-rich extrachromosomal circles (C-circles), and telomere sister chromatid exchange (T-SCE). Furthermore, LAP2α emerges as a crucial player in break-induced telomere replication for telomerase-positive cells following telomeric double-strand breaks. Mechanistically, our investigation suggests that LAP2α may influence the regulation of the heterochromatic state of telomeres, thereby affecting telomeric accessibility. In line with our findings, LAP2α expression is markedly reduced in ALT-positive osteosarcoma. And the use of methotrexate (MTX) can restore the heterochromatin state altered by LAP2α depletion. This is evidenced by a significant inhibition of tumor proliferation in ALT-positive patient-derived xenograft (PDX) mouse models. These results indicate the important role of LAP2α in regulating ALT activity and offer insights into the interplay between lamina-associated proteins and telomeres in maintaining telomere length. Importantly, our findings may help identify a more appropriate target population for the osteosarcoma therapeutic drug, MTX.

## Introduction

Telomere is a nucleoprotein structure found at the end of the linear chromosome, which is composed of TTAGGG repetitive DNA and shelterin complex. Human shelterin is a six-subunit complex (TRF2, TRF1, RAP1, TPP1, TIN2, and POT1) that binds telomeres and shields the ends of chromosomes from degradation and end-to-end fusions [1]. Due to the terminal replication problem, the length of the telomere gradually shortened with continuous cellular proliferation, and the cells will eventually undergo cell cycle arrest and then cellular senescence [2]. Activation of the telomere maintenance mechanism to prevent excessive telomere shortening is required for the sustained proliferation in cancer cells [3]. Most cancers use telomerase to extend their telomeres [4], but 10–15% of cancers elongate their telomere through telomerase independent pathway, which is named alternative lengthening of telomeres (ALT) [5, 6].

ALT was first found in the telomerase mutational budding yeast [7], and then it was observed in human tumors. In the past decades, researchers have done a lot of work to uncover the molecular mechanism of ALT. Even though the precise molecular mechanism of ALT is still unclear, it is widely accepted that ALT is a DNA repair process dependent homologous recombination process [8], which is represented by the appearance of a variety of hallmarks, including extra chromosome telomere repeat (CTR, such as C-circle), ALT-associated PML bodies (APBs), heterogeneity of telomere length, and telomere sister chromatid exchange (T-SCE) [5]. In addition, accumulating evidence indicates that the long noncoding RNA (lncRNA) telomeric repeat-containing RNA (TERRA) is elevated in ALT cells [9, 10].

The current studies revealed that the presence of recombinase RAD51 and RAD52 is essential for ALT telomere maintenance [11]. Inhibition of RAD52 decreases the natural ALT telomere synthesis at APBs in G2 cells [12], and the depletion of RAD51 leads to an increase of fragile telomeres and telomere dysfunction-induced foci in ALT cells but does not affect the mitotic DNA synthesis (MiDAS) at telomeres [13]. Instead of directly taking part in ALT DNA homologous recombination, RAD51 may mainly function in lessening telomere fragility by suppressing the stalled replication forks. Moreover, RAD52-mediated telomeric MiDAS is observed in both ALT-positive and telomerase-positive cells when cells encounter exogenous DNA damage or replication inhibitor, indicating that RAD52 is essential for break-induced replication (BIR) at collapsed forks and fragile sites. Interestingly, a most recent study demonstrates that the C-circle levels are not altered in RAD52 knockdown cells, suggesting that there is a RAD52-independent ALT pathway, which is responsible for C-circle generation during telomere damage [14]. Meanwhile, POLD3/POLD4 is reported to be required for conservative DNA replication during BIR, which could be enhanced by BLM and reduced by SLX4 [15]. In ALT-positive human cells, telomeres undergo conservative synthesis and also rely on POLD3/4, thereby linking ALT to BIR.

53BP1 nuclear bodies are formed when DNA damage occurs in the S phase. However, it is excluded from DNA breaks during mitosis. In contrast, the ssDNA-binding protein RPA can bind to exposed ssDNA in M-phase [16]. Recent research reported that RPA plays a critical role in protecting resected ssDNA upon chromosome breakage and promoting the successful progress of telomeric MiDAS [17]. In addition, the elevated level of TERRA suggests it plays a crucial role in ALT telomeres recombinogenic by forming R-loop or RNA–DNA hybrids with the telomeric C-rich DNA strand, which contributes to the activation of the DNA damage response (DDR) and promotes recruitment of the recombinase [18, 19]. The aberrant accumulation of phosphorylated RPA at ALT telomeres when the RNaseH1 was deleted, which regulates the levels of RNA–DNA hybrids between telomeric, indicates that RPA itself could promote the forming of R-loop. Supporting this, it has been reported that the C-rich telomeric ssDNA accumulated in ALT cells when the RPA was depleted [20]. Moreover, in yeast, the telomeres are bound by the Rap1 protein. Rap1 interacts with Sir3 and Sir4, which, together with the histone deacetylase Sir2, initiate heterochromatin formation in subtelomeric regions [21]. The Sir2/Sir3/Sir4 complex also restricts TERRA expression via transcriptional repression [22]. Therefore, the roles of RPA and RAP1 in TERRA R-loop regulation in human ALT cells deserve further exploration.

Previous studies have shown that Lamin A/C is involved in DNA damage repair and telomere maintenance [23–26]. As a lamina-associated protein, lamina-associated polypeptide 2(LAP2) belongs to the LEM domain protein family. There are six splicing isoforms encoded by the *TMPO*, including LAP2 α, β, γ, δ, ε, and ζ [27]. All isoforms have an LEM domain and LEM-like domain, by which to interact with BAF protein and bind to DNA [28, 29]. Unlike the other isoforms, LAP2α and ζ lack a transmembrane domain; thus, they could only localize at nucleoplasm with Lamin A/C [30, 31]. In addition, it is reported that LAP2α interacted with WRN helicase and Ku86 [25], which are essential for classical nonhomologous end-joining repair and telomere integrity during telomeric DNA damage repair [32–34]. Moreover, it has been recently reported that LAP2α could promote the deposition of RPA on damaged DNA and facilitate homologous recombination (HR) [35]. It has been demonstrated that the distribution of LAP2α in cells changes with cell cycles [36], and it binds to the telomere or sub-telomere at the anaphase and telophase of mitosis [37]. Additionally, it has demonstrated that LAP2α could located at the end of chromosome and colocalized with telomere during anaphase and telophase of mitosis. Thus, we speculate that LAP2α might play a critical role in break-induced replication-mediated alternative telomere lengthening.

In this study, we demonstrated the interaction of LAP2α with the shelterin complex. The knockdown of LAP2α resulted in a significant increase in ALT-associated hallmarks, including APBs, C-circles, and T-SCE, emphasizing LAP2α‘s crucial role in ALT maintenance. Subsequent investigations unveiled that LAP2α depletion led to the recruitment of recombination factors, such as RPA and RAD52, to telomeres. Moreover, LAP2α was found to interact with HDAC1, potentially promoting the heterochromatin state of telomeric DNA, inhibiting telomere recombination and ALT occurrence. This observation aligns with our findings that LAP2α expression is low in ALT-positive osteosarcoma patients. Furthermore, methotrexate (MTX), a widely used drug for treating osteosarcoma, has been reported to enhance DNA heterochromatinization. Our discovery revealed that MTX treatment increased the aggregation of H3K9 trimethylation and HP1 in the telomeric region. Notably, in our patient-derived xenograft (PDX) model, MTX exhibited specific inhibition of the proliferation of LAP2α-low-expressing ALT-positive osteosarcoma tumors. Mechanistically, our data provide initial evidence that the lamina-associated protein LAP2α intricately participates in ALT telomere synthesis by regulating telomeric heterochromatin status. This breakthrough opens new avenues for unraveling the precise molecular mechanisms underlying ALT, offering promising insights for future investigations. Importantly, our findings enhance the targeted use of MTX, providing a critical context for its improved clinical performance.

## Results

### LAP2α interacted with shelterin complex and LAP2α deficiency induced dysfunctional telomeres of ALT-positive cell

**Fig. 1:**
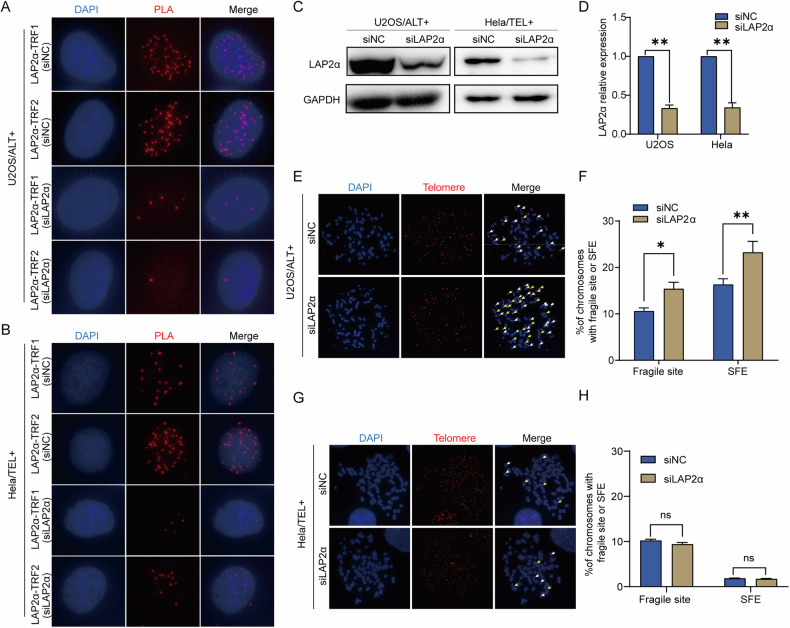
LAP2α interacted with shelterin complex and LAP2α deficiency induced dysfunctional telomeres of ALT-positive cells. **A**, **B** PLA of LAP2α and subunit of shelterin complex in U2OS cells and Hela cells transfected with control or LAP2α siRNA. Red dots represent PLA signals. **C** U2OS and Hela cells were infected with control or LAP2α siRNA for 3 days. Cell lysates were subjected to western blot analysis with anti-LAP2α and anti-GAPDH antibodies. GAPDH was used as the loading control. **D** Quantification of the LAP2α relative protein level in comparison to GAPDH and to siNC was calculated by ImageJ software. Error bars represent the mean ± SEM of four independent experiments. Two-tailed unpaired Student’s *t*-test was used to calculate *p*-values. **p < 0.01. **E**, **G** Metaphase chromosome spreads and telomere FISH in U2OS and Hela cells. Representative images showing fragile sites (yellow arrows) and signal-free ends (SFE, white arrows). **F**, **H** Quantification of the fragile site and SFEs of U2OS and Hela cells. Error bars represent the mean ± SEM of three independent experiments. Two-tailed unpaired Student’s *t*-test was used to calculate *p*-values. ns, not significant or *p* ≥ 0.05; *p < 0.05; **p < 0.01.

Previous research has suggested that LAP2α, a lamin-associated protein, may participate in nuclear membrane reconstruction during mitosis and localize to the end of chromosomes during anaphase and telophase [36]. This led us to investigate whether LAP2α was necessary for telomere maintenance. We initially validated the interaction between LAP2α and telomeres through proximity ligation assays (PLA), confirming the association between LAP2α and telomere-binding proteins TRF1 and TRF2. This assessment was conducted in both ALT-positive U2OS and SAOS2 cells, as well as telomerase-positive HeLa cells (Fig. 1A, B and Supplementary Fig. S1A). PLA foci were present in all three cell lines, and the knockdown of LAP2α in these cell lines significantly reduced the PLA foci. These findings suggest that LAP2α interacts with the shelterin complex in vivo, regardless of whether the cells are ALT-positive or telomerase-positive. We then tested whether LAP2α plays a role in telomere maintenance by transiently transfecting cells with LAP2α siRNA (Fig. 1C, D) and examining telomere defects, including fragile telomere formation and telomere loss, in metaphase cells. Our results showed that LAP2α knockdown in U2OS cells significantly increased the number of fragile sites on telomeres and the number of chromosome ends lacking telomere FISH signals (Signal free end, SFE) compared to siNC controls (Fig. 1E, F). However, we did not observe any effect of LAP2α knockdown on telomere maintenance in HeLa cells (Fig. 1G, H). To further confirm the non-specific role of LAP2α in osteosarcoma cells, telomerase-positive osteosarcoma cells MG32 were utilized. Even in MG32 cells, we did not observe any impact of LAP2α knockdown on telomere maintenance (Supplementary Fig. S1B, C). These findings suggest that, despite LAP2α interacting with telomeres in multiple cell types, its crucial role in telomere maintenance appears to be primarily in ALT cells and may not occur in telomerase-positive cells.

### Knockdown of LAP2α elevates the telomeric homologous recombination and extends the telomere length of ALT cell

**Fig. 2:**
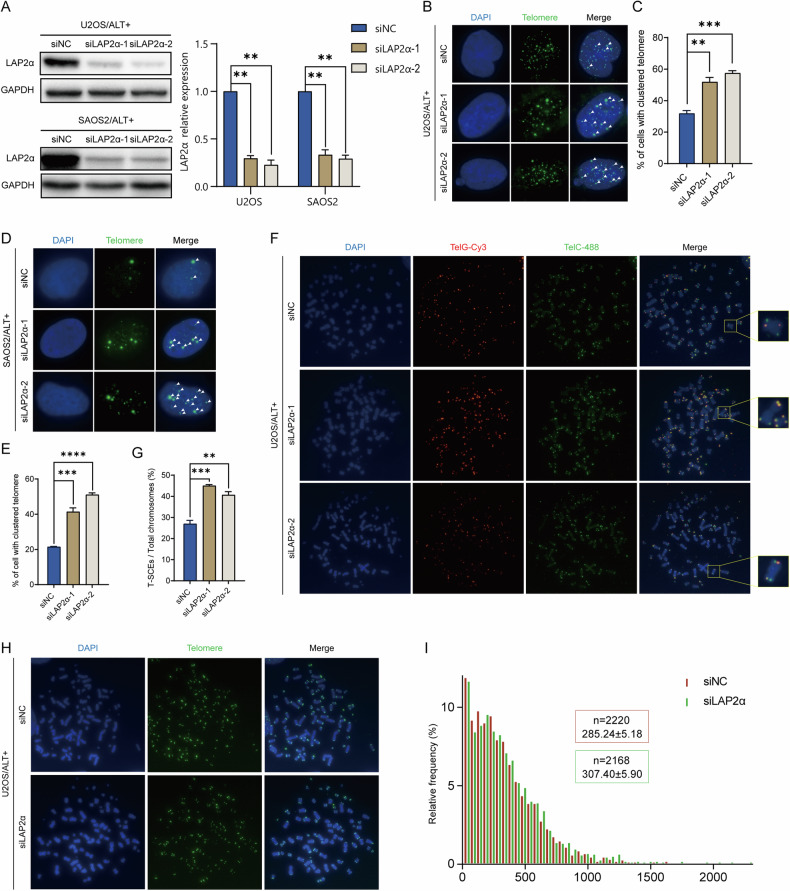
Knockdown LAP2α elevates the telomeric homologous recombination and extends the telomere length of ALT cells. **A** Left panel: LAP2α expression was measured by western blot after infecting with control or LAP2α siRNA in U2OS and SAOS2 cells. Right panel: Quantification of the LAP2α relative protein level in comparison to GAPDH and to siNC was calculated by ImageJ software. Error bars represent the mean ± SEM of four independent experiments. Two-tailed unpaired Student’s *t*-test was used to calculate *p*-values. **p < 0.01. **B** Representative images showing telomere clustering of U2OS cells in the presence of control or LAP2α siRNA. **C** Quantification of (**B**), the percentage of cells containing clustered telomeres. Error bars represent the mean ± SEM of three independent experiments. Two-tailed unpaired Student’s *t*-test was used to calculate *p*-values. **p < 0.01; ***p < 0.001. **D** Representative images showing telomere clustering of SAOS2 cells. **E** Quantification of (**D**). Error bars represent the mean ± SEM of three independent experiments. Two-tailed unpaired Student’s *t*-test was used to calculate *p*-values. ***p < 0.001;****p < 0.0001. **F** Telomeric DNA CO-FISH on metaphases from U2OS cells to reveal T-SCEs. Cells were harvested 3 days after siRNA transfection. **G** Quantification of the average number of T-SCEs per chromosome detected in U2OS cells. Error bars represent the mean ± SEM of three independent experiments. Two-tailed unpaired Student’s *t*-test was used to calculate *p*-values. **p < 0.01; ***p < 0.001. **H** Q-FISH analysis of relative telomere length in U2OS cells. Representative images showing telomeres hybridized with PNA Alexa 488-labeled telomere probe (green). **I** Frequency distributions of relative telomere length telomere length of (**H**).

Next, we aimed to further understand the role of LAP2α in ALT cells. Mechanistically, ALT is a telomere elongation mechanism that relies on homology-directed repair [38, 39], which leads to telomere aggregation termed “telomere clustering” [40]. Therefore, we examined the effect of LAP2α disruption on telomere clustering in ALT cells. U2OS and SAOS2 cells were transfected with two different LAP2α siRNAs for 72 h (Fig. 2A), and the percentage of cells with clustered telomeres was determined. The results showed that the percentage of U2OS (Fig. 2B, C) and SAOS2 (Fig. 2D, E) cells with telomere clustering significantly increased upon LAP2α depletion. To directly observe telomeric homologous recombination in ALT cells, telomere chromosome orientation fluorescence in situ hybridization (CO-FISH) was carried out, and the occurrence of telomere sister chromatid exchange (T-SCE) events was determined. Consistent with the results of telomere clustering, the frequency of chromatid exchange was significantly increased in LAP2α-knockdown U2OS cells (Fig. 2F, G). These findings support the idea that LAP2α inhibits telomeric homologous recombination in ALT cells.

Furthermore, we estimated telomere length by quantitative-FISH (Q-FISH) using Alexa 488-labeled or Cy3-labeled PNA probes. The average fluorescence unit (AFU) was used to reflect relative telomere length. Our results showed that the telomere length was slightly extended when LAP2α was knocked down with both probes (Fig. 2H, I and Supplementary Fig. S2A, B).

### LAP2α depletion stimulates the formation of hallmarks of ALT

**Fig. 3:**
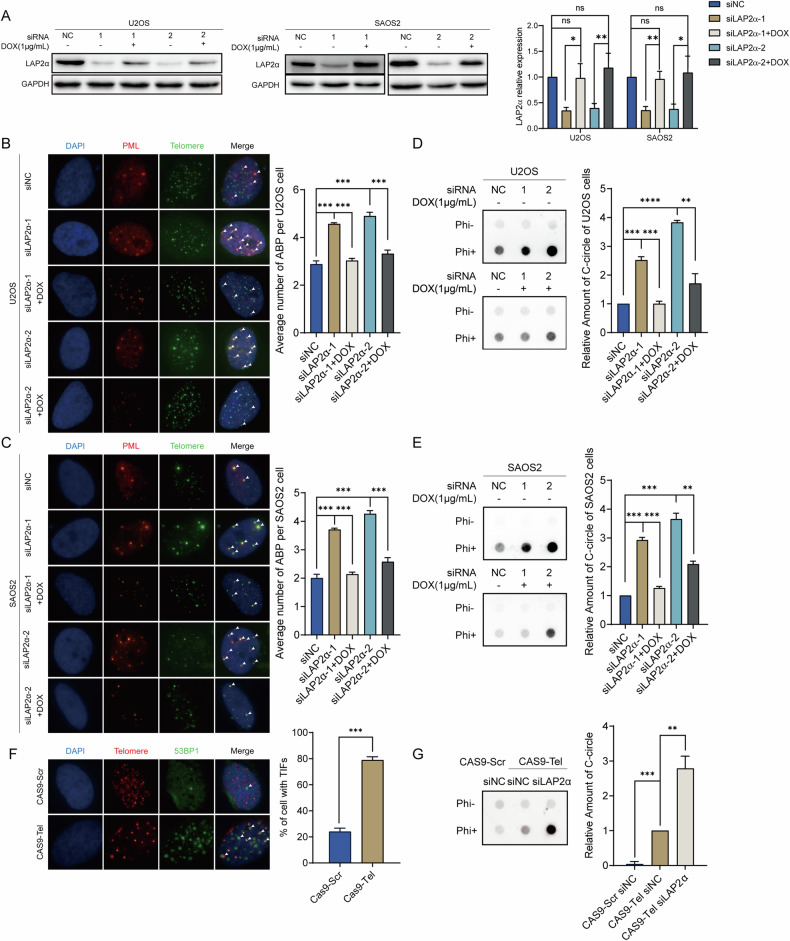
LAP2α depletion stimulates the formation of hallmarks of ALT. **A** Left panel: Western blot analyzes the expression of LAP2α in U2OS (left) and SAOS2 (right) cells transiently transfected with control siRNA or LAP2α siRNA for 24 h and then re-expressing doxycycline (DOX)-inducible RNAi-resistant LAP2α for 48 h. Right panel: Quantification of the LAP2α relative protein level in comparison to GAPDH and to siNC was calculated by ImageJ software. Error bars represent the mean ± SEM of four independent experiments. Two-tailed unpaired Student’s *t*-test was used to calculate *p*-values. ns, not significant or *p* ≥ 0.05; *p < 0.05; **p < 0.01. **B** Representative images show the colocalization of telomere (green, detected by FISH) and PML (red, detected by immunofluorescence) to measure ALT-associated PML bodies (APBs) formation upon LAP2α knockdown in U2OS cells (left panel). Quantification of the average number of APBs in the U2OS cells (right). Error bars represent the mean ± SEM of three independent experiments. Two-tailed unpaired Student’s *t*-test was used to calculate *p*-values. ***p < 0.001. **C** The APBs formation upon LAP2α knockdown in SAOS2 cells (left panel). Quantification of the average number of APBs (right panel). Error bars represent the mean ± SEM of three independent experiments. Two-tailed unpaired Student’s *t*-test was used to calculate *p*-values. ***p < 0.001. **D** C-circle assay in ALT-positive cell lines U2OS cells (left panel). Quantification of the average amount of C-circle (right panel). Error bars represent the mean ± SEM of three independent experiments. Two-tailed unpaired Student’s *t*-test was used to calculate *p*-values. **p < 0.01; ***p < 0.001. **E** C-circle assay in ALT-positive cell lines SAOS2 cells (left panel). Quantification of the average amount of C-circle (right panel). Error bars represent the mean ± SEM of three independent experiments. Two-tailed unpaired Student’s *t*-test was used to calculate *p*-values. **p < 0.01; ***p < 0.001. **F** The colocalization of telomere (red, FISH) and 53BP1 (green, IF) to measure dsDNA breaks at telomere generated by the CRISPR/Cas9 system (left panel). Error bars represent the mean ± SEM of three independent experiments. Two-tailed unpaired Student’s *t*-test was used to calculate *p*-values. ***p < 0.001. Quantification of the percentage of cells with TIFs (right panel). **G** C-circle assay in Hela cells (left panel). Quantification of the average amount of C-circle (right panel). Error bars represent the mean ± SEM of three independent experiments. Two-tailed unpaired Student’s *t*-test was used to calculate *p*-values. **p < 0.01; ***p < 0.001.

Although the precise molecular mechanism of ALT remains unclear, there are several hallmarks of ALT, including the formation of APBs and C-circles. To better understand the function of LAP2α in the ALT pathway, we examined the formation of APBs by determining the colocalization of PML and telomeric DNA in LAP2α knockdown U2OS and SAOS2 cells. The results showed that the average number of APBs per cell was significantly increased upon LAP2α removal (Fig. 3A–C). Consequently, a rescue experiment was conducted by re-expressing the doxycycline (DOX)-inducible RNAi-resistant LAP2α in the knockdown cell lines, and we observed that the recovery of LAP2α restrained the formation of APBs in both cell lines (Fig. 3A–C). In addition, upon LAP2α depletion, the formation of C-circles was significantly increased in two ALT cell lines, and subsequent rescue experiments further confirmed that LAP2α depletion stimulates the ALT process (Fig. 3D, E). Meanwhile, LAP2α was knocked down in telomerase-positive HeLa cells and telomerase-positive osteosarcoma cells MG32, and the formation of C-circles was examined. We found that LAP2α deficiency alone could not be enough to initiate the formation of C-circles (Supplementary Fig. S3A). These findings confirm that LAP2α is essential for ALT maintenance, but the knockdown of LAP2α alone is not sufficient to initiate ALT in telomerase-positive cells.

Previous reports have shown that sustained double-strand breaks (DSBs) at telomeric DNA can lead to the formation of ALT-like characteristics and result in the initiation of recombination and elongation of telomeres in telomerase-positive cell lines [15, 41, 42]. Thus, we attempted to deliberate the role of LAP2α in telomerase-positive cells upon telomeric DNA breaks. First, double-strand telomeric DNA breaks were induced in HeLa cells using the CRISPR-CAS9-sgTel system, which consists of sgRNA targeting telomeric DNA as previously reported [43]. Immunofluorescence (IF) and fluorescence in situ hybridization (FISH) were performed to visualize the localization of 53BP1, an indicator of DNA damage response, with telomeres to verify the formation of telomeric-specific breaks. As expected, 53BP1 was recruited to telomeres to form telomere dysfunction foci (TIF) in HeLa cells after infection with CAS9-sgTel lentivirus (Fig. 3F). And the hallmarks of ALT, such as the formation of APBs and C-circles, were induced in HeLa cells upon treatment with CAS9-sgTel lentivirus (Supplementary Fig. S3B–D). Moreover, when we knocked down LAP2α after telomeric DNA breaks were induced, the formation of C-circles caused by CAS9-sgTel was significantly exacerbated (Fig. 3G). This finding again proves that LAP2α is crucial for telomeric break-induced repair and the initiation of telomere recombination in telomerase-positive cells upon breaks.

### LAP2α repressing the recruitment of recombination factors to telomeres

**Fig. 4:**
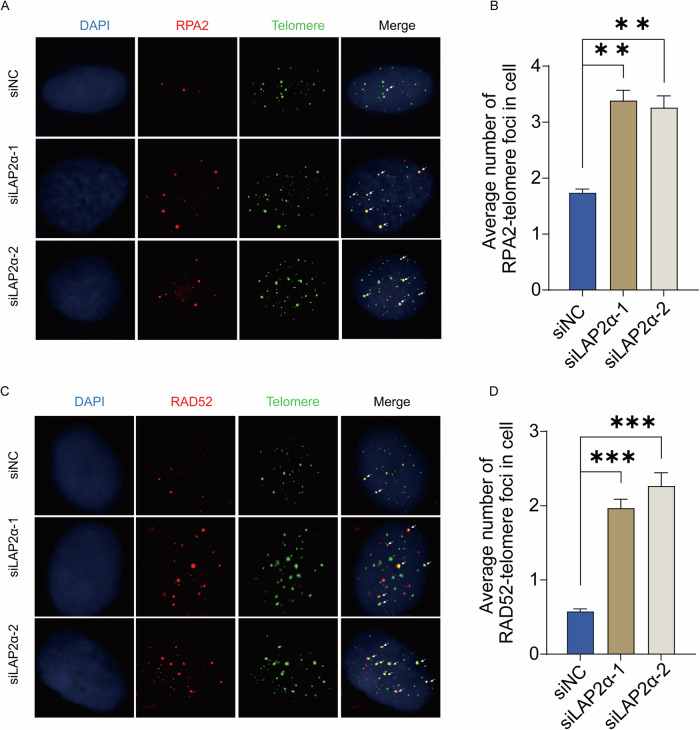
LAP2α repressing the recruitment of recombination factors to telomeres. **A** Analysis of the colocalization of telomere (G-rich probe, green) and RPA2 (antibodies, red) by IF-FISH in U2OS cells were transiently transfected with control siRNA or LAP2α siRNA for 72 h. **B** Quantification of panel (**A**). Error bars represent the mean ± SEM of three independent experiments. Two-tailed unpaired Student’s *t*-test was used to calculate *p*-values. **p < 0.01. **C** Analysis of the colocalization of telomere (green) and RAD52 (red) by IF-FISH in U2OS cells. **D** Quantification of panel (**C**), respectively. Error bars represent the mean ± SEM of three independent experiments. Two-tailed unpaired Student’s *t*-test was used to calculate *p*-values. ***p < 0.001.

RPA (replication protein A), the sensor of single-stranded DNA (ssDNA) that plays an essential role in DNA damage repair and homologous recombination, has been shown to colocalize with telomeric DNA in human ALT cells [44, 45]. Therefore, we examined the recruitment of RPA to telomeres by IF-FISH when LAP2α was knocked down. We observed a significant increase in the colocalization of RPA2 with telomeres upon LAP2α depletion (Fig. 4A, B). Furthermore, RPA is crucial for the assembly of RAD52 recombinase during DNA double-strand break repair [46, 47], and RAD52 plays an important role in homologous telomere elongation in ALT cells [12, 14, 48]. Therefore, we also examined the recruitment of RAD52 to telomeres upon LAP2α depletion. Similarly, the recruitment of RAD52 to telomeres in U2OS cells increased after LAP2α knockdown (Fig. 4C, D). We were unable to detect any foci of Rad51, therefore, we could not determine the colocalization of Rad51 and telomeres (Supplementary Fig. S4). Taken together, these findings indicate that the absence of LAP2α promotes the recruitment of recombination factors, including RPA and Rad52, to telomeres, thereby facilitating the ALT process.

### Lack of LAP2α decreased the heterochromatin status of telomere

**Fig. 5:**
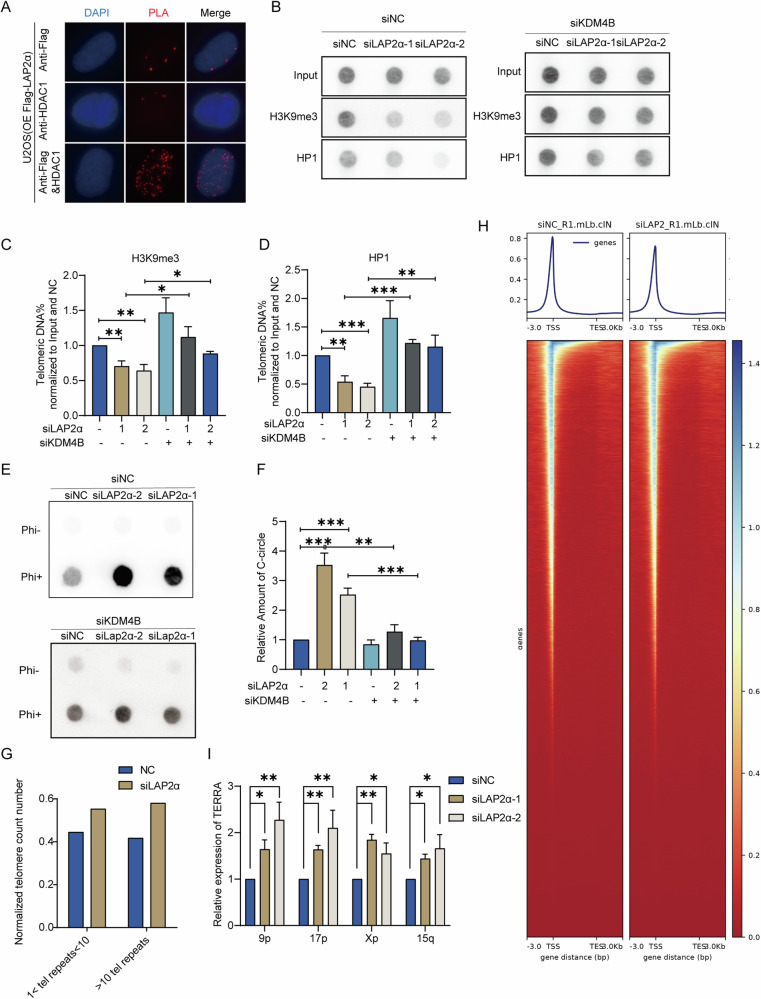
Lack of LAP2α decreased the heterochromatin status of telomere. **A** PLA assay was used to analyze the interaction between LAP2α and HDAC1 in U2OS over-expressing Flag-LAP2α. Red dots represent PLA signals. **B** U2OS cells were transiently transfected with control siRNA or LAP2α siRNA for 48 h and then transfected with control siRNA or KDM4B siRNA for 48 h, cells were harvested and subjected to ChIP experiments using antibodies raised against trimethylated H3K9, HP1 and control IgG binding to telomeres. Telomere repeat DNA was visualized by dot blot and probed with a telomere-specific probe. **C**, **D** Quantification of H3K9me3 and HP1 binding to telomeres at least three different ChIP assays represented by panel (**B**). Error bars represent the mean ± SEM of three independent experiments. Two-tailed unpaired Student’s *t*-test was used to calculate *p*-values. *p < 0.05; **p < 0.01; ***p < 0.001. ChIP DNA signals were normalized to input DNA signal and to siNC-treated cells. **E** C-circle assay in U2OS cells transfected with LAP2α siRNA and/or KDM4B siRNA. **F** Quantification of the relative amount of C-circle. Error bars represent the mean ± SEM of three independent experiments. Two-tailed unpaired Student’s *t*-test was used to calculate *p*-values. **p < 0.01; ***p < 0.001. **G** Copy number normalized ATAC-seq counts mapped to telomere regions. **H** Average profiles of ATAC-seq peaks in control siRNA or LAP2α siRNA treated U2OS cells at transcription start site (TSS) ± 3 kb (top). Heatmap of densities of ATAC-Seq peaks of genes in control siRNA or LAP2αsiRNA treated U2OS cells (bottom). **I** qRT-PCR analysis of TERRA levels in the indicated U2OS cells. TERRA levels were detected using primers specific for TERRA transcribed from subtelomeres of human chromosome 9P, 17P, Xp and 15q, as indicated. Error bars represent the mean ± SEM of three independent experiments. Two-tailed unpaired Student’s *t*-test was used to calculate *p*-values. *p < 0.05; **p < 0.01. Data are represented as mean ± SEM of three or more independent experiments, and *p < 0.05; **p < 0.01; ***p < 0.001 (Student’s *t*-test).

The integrity of telomeric heterochromatin is thought to be a suppressor of ALT, given that the highly condensed chromatin structure inhibits recombination processes [49, 50]. Loss of nuclear lamina proteins, such as Lamin A/C [51, 52] and SUN [53], can alter the degree of DNA heterochromatinization. Additionally, LAP2α has been documented to interact with histone deacetylases (HDAC1), reinforcing the role of heterochromatin in chromatin condensation [54, 55]. Therefore, our subsequent investigation delved into the interaction between LAP2α and HDAC1, alongside the assessment of histone epigenetic modifications at the telomere region following LAP2α depletion. Proximity ligation assay (PLA) results demonstrated the in vivo interaction between LAP2α and HDAC1 (Fig. 5A). Moreover, LAP2α disruption led to a ~30–40% reduction in H3K9 trimethylation and altered HP1 localization at telomeric repeats (Fig. 5B–D), suggesting that LAP2α depletion may impede the recruitment of HDAC1 to telomeres, consequently inducing telomeric chromatin decondensation. It has been reported that mutations in the demethylase KDM4B can enhance DNA heterochromatinization and reduce telomeric accessibility. This alteration appears to correlate with increased levels of heterochromatin-associated proteins, including H3K9me3, ATRX, and HP1, observed in KDM4B knockout cells. To further validate the role of LAP2α in driving the formation of Alternative Lengthening of Telomeres (ALT) through the regulation of telomeric DNA heterochromatin status, KDM4B was depleted in siLAP2α U2OS cells to increase heterochromatinization in the telomeric region. Subsequently, its impact on ALT formation was elucidated by assessing C-circle formation. The current results indicate that the levels of H3K9me3 and HP1 at telomeres increased upon KDM4B knockdown in both siNC and siLAP2α U2OS cell lines (Fig. 5B–D). Furthermore, the heightened C-circle formation induced by LAP2α knockdown was rescued by depleting KDM4B (Fig. 5E, F). These findings suggest that LAP2α may play a significant role in influencing the formation of Alternative Lengthening of Telomeres (ALT) through its potential regulation of telomeric DNA heterochromatin status. To further investigate the impact of LAP2α on chromatin accessibility, we performed ATAC-seq analysis targeting the telomere region. Strikingly, our results demonstrated a significant increase in the number of accessible telomeres upon LAP2α depletion, providing further evidence for the stimulation of telomere accessibility by LAP2α disruption (Fig. 5G). Moreover, we explored the effect of LAP2α depletion on the accessibility of other genomic regions, including the 5’-UTR, 3’-UTR, gene body, and TSS. Remarkably, we observed a significant suppression of accessibility following LAP2α depletion, suggesting that LAP2α may exert a distinct regulatory function in modulating accessibility at various genomic loci (Fig. 5H). TERRA, transcribed from telomeric repeat-containing RNA, was reported to be down-regulated by the heterochromatic status of telomeric DNA [56]. To further confirm the role of LAP2α in regulating the heterochromatin state, we detected TERRA transcripts from individual chromosomes (including 9p, 17p, and 5q) and found that the TERRA transcribed from these chromosomes was significantly elevated in LAP2α knockdown U2OS cells (Fig. 5I) and SAOS2 cells (Supplementary Fig. S5). Taken together, our data suggest that LAP2α knockdown might lead to a reduction in telomeric heterochromatin, which could promote telomeric accessibility and the occurrence of ALT, possibly through the modulation of deacetylase HDAC1 recruitment to chromosome ends. However, further investigation is required to fully establish these mechanisms.

### LAP2α expression is lower in ALT-positive osteosarcoma (OS) and associated with poorer survival

**Fig. 6:**
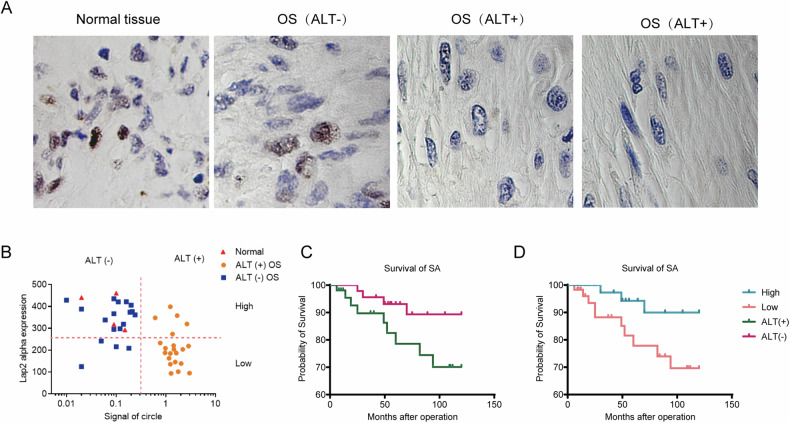
LAP2α expression is lower in ALT-positive osteosarcomas (OS) and associated with poorer survival. **A** Immunohistochemical staining with antibodies to LAP2α was performed on tumor and normal tissue from OS patients. **B** Quantitation of LAP2α staining and C-circle signal in OS (ALT+ and ALT−) and normal tissues from patients. **C** Kaplan–Meier metastasis-free survival curve for osteosarcoma patients showing the impact of ALT− or ALT+ on outcome. **D** Kaplan–Meier curves of overall survival for osteosarcoma patients based on LAP2α expression.

To further explore the LAP2α expression and its impact on ALT-positive tumor outcomes, we collected data from 39 OS cases, including 21 ALT-positive and 12 ALT-negative cases (Supplementary Fig. S6). Through immunohistochemistry, we analyzed LAP2α expression in 33 osteosarcoma tissues and 4 normal tissues (Fig. 6A). Expression levels were grouped as high or low based on a predetermined cut-off, and the C-circle signal distinguished ALT-negative and ALT-positive cases. Our analysis revealed significantly lower LAP2α expression in ALT-positive OS compared to ALT-negative OS and normal tissue (Fig. 6B). Kaplan–Meier analysis demonstrated poorer overall survival in ALT-positive cases (Fig. 6C) and better outcomes with increased LAP2α expression (Fig. 6D). Taken together, our findings indicate that the downregulation of LAP2α may be associated with the aggressive behavior of ALT-positive osteosarcoma, suggesting its potential as a marker or therapeutic target, although further studies are necessary to confirm its clinical relevance.

### Methotrexate reverses LAP2α-induced telomeric chromatin decondensation, offering basis for precise treatment of osteosarcoma

**Fig. 7:**
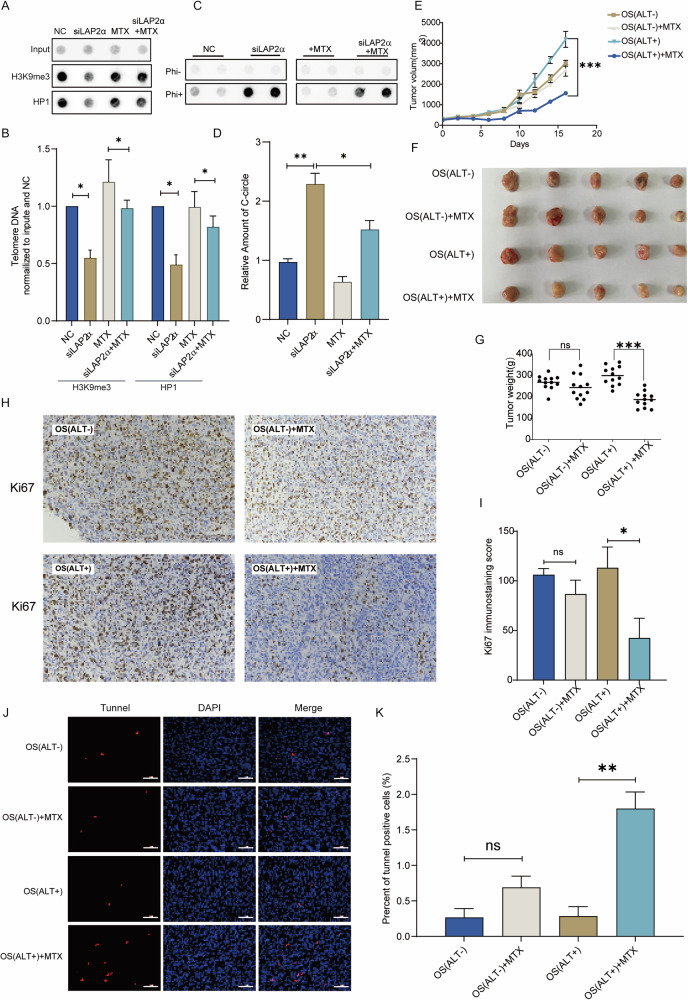
Methotrexate reverses LAP2α-Induced telomeric chromatin decondensation, offering basis for precise treatment of osteosarcoma. **A** U2OS cells were transiently transfected with control siRNA or LAP2α siRNA and/or treated with MTX, cells were harvested and subjected to ChIP experiments using antibodies raised against trimethylated H3K9, HP1 and control IgG binding to telomeres. Telomere repeat DNA was visualized by dot blot and probed with a telomere-specific probe. **B** Quantification of H3K9me3 and HP1 binding to telomeres. Error bars represent the mean ± SEM of three independent experiments. Two-tailed unpaired Student’s *t*-test was used to calculate *p*-values. *p < 0.05. **C** C-circle assay in U2OS cells treated with LAP2α siRNA and/or MTX. **D** Quantification of the amount of C-circle in (**C**). Error bars represent the mean ± SEM of three independent experiments. Two-tailed unpaired Student’s *t*-test was used to calculate *p*-values. *p < 0.05; **p < 0.01. **E** PDX mouse model implanted with a human ALT+ or ALT− osteosarcoma tumor was divided into 4 groups (12 mice per group) and treated with vehicle, 0.5 mg/kg MTX for 2 weeks, Treatment with MTX inhibits ALT+ osteosarcoma PDX tumor size compared with the vehicle group. Error bars represent the mean ± SEM of three independent experiments. Two-tailed unpaired Student’s *t*-test was used to calculate *p*-values. ***p < 0.001. **F** ALT+ PDX tumor morphology decreased after treatment with MTX compared to the control group. MTX did not significantly affect ALT− PDX tumor morphology compared with the vehicle group. **G** The graph of PDX tumor weight: ALT+ PDX tumor weight in the group treated with MTX was lower than the other groups. Error bars represent the mean ± SEM of three independent experiments. Two-tailed unpaired Student’s *t*-test was used to calculate *p*-values. ns, not significant; ***p < 0.001. **H** Representative images of immunohistochemistry (IHC) staining for Ki67 (×20). **I** The analysis of Ki67 positive cells. **J** The representative Tunnel (red) and DAPI (blue) staining (×20) results of tumor tissues from PDX-model mouse in different groups after MTX treatment. **K** The relative Tunnel positive rates (%) in different groups. Note: Two-tailed unpaired Student’s *t*-test was used to calculate *p*-values. ns, not significant; **p < 0.01.

As previously mentioned, the knockdown of LAP2α has been associated with the promotion of ALT by decondensing telomeric chromatin. Given that methotrexate (MTX), a commonly used chemotherapy drug for osteosarcoma recently been identified as a heterochromatin-promoting agent by increasing the level of H3K9me3 [57], we aimed to investigate whether MTX could restore telomeric chromatin decondensation caused by LAP2α knockdown. Chromatin immunoprecipitation (ChIP) was performed, and telomeric heterochromatin markers H3K9me3 and HP1 were detected using dot blot analysis. The results demonstrated that the decrease in H3K9me3 and HP1 induced by LAP2α depletion was restored by MTX treatment (Fig. 7A, B), indicating the capacity of MTX treatment to counteract the decreased heterochromatinization induced by the knockdown of LAP2α. Subsequently, the C-Circle generation was determined. We observed that the C-Circle signal was also reduced by ~34.5% after MTX treatment compared to the LAP2α depleted cells (Fig. 7C, D). This observation provides further support for the idea that LAP2α plays a crucial role in influencing the occurrence of ALT and that MTX can modulate this mechanism.

Given the subdivision of osteosarcoma into ALT+ and ALT− categories, where ALT+ exhibits a more open chromatin level in the telomere region compared to ALT−, we sought to determine if methotrexate exhibits differential sensitivity in these subtypes, aiming to offer improved guidance for clinical medication. To explore this, we established an osteosarcoma tumor patient-derived xenograft (PDX) mouse model, successfully generating one ALT-positive PDX(OS(ALT+)) group and one ALT-negative PDX(OS(ALT−)) group. Subsequently, tumor tissues were extracted, dissociated into cells, and then re-implanted into NSG mice to establish a second generation of PDX mice. The mice were divided into four groups (*n* = 12 mice per group) and subjected to treatment with either vehicle or 0.5 mg/kg/day MTX. Tumor volume and weight were closely monitored throughout the course of MTX treatment. These results indicated a significant reduction in OS(ALT+) tumor growth following MTX treatment compared to the vehicle-treated group (Fig. 7E). Intriguingly, methotrexate exhibited no observable impact on OS(ALT−) tumor growth relative to the vehicle-treated group (Fig. 7F, G).

Subsequently, we evaluated the impact of MTX on cell proliferation in the two different OS groups by assessing overall Ki67 staining levels through immunohistochemistry. As shown in Fig. 7H, I, both the OS(ALT−) and OS(ALT+) groups exhibited high Ki67 staining levels. In contrast, a significant reduction in Ki67 staining was observed in the OS(ALT+)+MTX group compared to the OS(ALT+) group. This suggests that MTX may effectively suppress the proliferative capacity of ALT-positive OS cells, as reflected by the decreased Ki67 staining intensity, which is consistent with the results from the tumor formation experiments (Fig. 7E, F). Next, to further elucidate the toxic mechanisms of MTX on ALT+ OS tumors, we assessed telomere damage, cell apoptosis, and cellular senescence. Telomere damage was first evaluated by analyzing telomere dysfunction-induced foci (TIFs) through the immunofluorescence and FISH colocalization of 53BP1 with telomeres. The data revealed a significantly higher frequency of TIFs in the MTX-treated group compared to the vehicle-treated group. However, MTX induced an approximately 5-fold increase in TIFs in the OS(ALT+) group, while only about a 0.5-fold increase was observed in the OS(ALT−) group (Supplementary Fig. S7A, B), indicating that MTX causes more pronounced telomere damage in ALT+ cells. We then examined the effects of MTX on apoptosis in OS cells in vivo using TUNEL staining. As shown in Fig. 7I, J, the OS(ALT+)+MTX group (1.8 ± 0.40%) displayed a significantly higher level of apoptosis compared to the OS(ALT+) group (0.29 ± 0.23%) (*p* < 0.01). However, no statistically significant difference was observed in TUNEL-positive apoptotic cells between the OS(ALT−)+MTX group (0.963 ± 0.28%) and the OS(ALT−) group (0.27 ± 0.22%), suggesting that MTX selectively induces apoptosis in ALT-positive OS cells in vivo (Fig. 7I, J). Finally, p16 expression was assessed by immunohistochemistry to evaluate MTX-induced cellular senescence in OS cells within the PDX model. However, no significant increases in p16-positive cells were observed across all four groups (Supplementary Fig. S7C). These data suggest that MTX likely reduces the proliferation rate of ALT-positive cells by inducing telomere damage, leading to subsequent apoptosis. Collectively, these findings suggest that patients with the more aggressive ALT+ phenotype, characterized by lower LAP2α expression, may benefit from MTX treatment. Once again, this reaffirms that categorizing osteosarcoma based on the telomere extension pathway has the potential to enhance guidance for clinical medication decisions.

## Discussion

Telomeres and telomerase are targets for anticancer drug development, and specific inhibitors are currently being investigated. However, only a few telomerase inhibition approaches have been used in clinical settings, possibly due to the activation of the ALT pathway in response to persistent telomere DNA damage caused by these inhibitors, leading to increased tumor aggressiveness [58, 59]. Therefore, understanding the telomere maintenance mechanism underlying the transition between the telomerase-dependent and ALT pathway is critical. This study reveals that LAP2α interacts with telomeres (TRF1/TRF2), suppressing ALT by inhibiting recombination factor recruitment. The occurrence is likely associated with its interaction with HDAC1, which contributed to enhancement in heterochromatinization and reduced accessibility of telomere. In addition, the observation that LAP2α expression is increased in ALT-negative osteosarcoma patients further supports its significance in suppressing homologous recombination-mediated telomere lengthening. Patients with lower LAP2α expression in ALT-positive osteosarcoma exhibit a poorer prognosis and lower survival rates, prompting the consideration of whether attenuated heterochromatinization caused by LAP2α depletion can impede the growth of ALT tumors. Coincidentally, methotrexate (MTX), a common drug for osteosarcoma, is known to enhance heterochromatinization. The administration of MTX was observed to inhibit ALT occurrence and suppress the proliferation of LAP2α lower-expressed ALT-positive patient-derived xenograft (PDX) tumors. In contrast, its impact on ALT-negative OS tumors was not pronounced. To summarize, These findings offer valuable insights into the regulation of telomere maintenance and may have implications for the development of novel therapeutic strategies targeting telomere maintenance in ALT-positive cancers.

Until now, the detailed molecular mechanism of the ALT pathway has not been fully understood. It has been suggested that ALT maintains telomeres through a homology-directed repair (HDR) process, involving various recombination factors, such as RPA, RAD51/RAD52, BLM, and SLX4 [60]. Upon DNA damage, cells initiate a DNA damage response process, resulting in a temporary opening of the chromatin structure around the damaged sites. This facilitates the access of DNA repair complexes to the affected regions. ALT is considered a telomere break-induced replication process, requiring the decondensation of telomeric DNA [61, 62].

Typically, compacted heterochromatin is situated at the nuclear periphery, closely associated with the nuclear envelope (NE). Nuclear envelope proteins play roles in facilitating the attachment of telomere to the nuclear lamina (NL) [63]. Although LAP2α‘s effect on telomeres is not well-explored, studies have elucidated the significant role of lamins in telomere homeostasis [23, 64, 65]. Lamin A, for instance, interacts with TRF2, contributing to the stabilization of chromosome-end structures [66]. Diminished lamin A/C or LMNA mutations stimulate telomere-telomere recombination [67] and reducinterstitial telomere-loop formation [68]. Furthermore, the interaction between SUN1, a protein interacting with Lamin A, and RAP1 establishes a link between the nuclear envelope (NE) and the shelterin complex [37, 69]. SUN1/2 interactions with the DNA-dependent protein kinase (DNAPK) complex contribute to telomere nonhomologous end-joining repair [70]. Therefore, these collective insights shed light on the intricate interplay of nuclear envelope-associated molecular components in the regulation of telomeres’ integrity.

Notably, LAP2α expression has been observed to mitigate the LMNA mutations-induced loss of H3K27me3, an epigenetics modification typically associated with gene silencing and chromatin compaction, especially in the telomere region [71]. In addition, the colocalization of LAP2α with telomeres and H3K27me3 undergoes attenuation in Hutchinson-Gilford Progeria Syndrome (HGPS), a condition caused by mutations in the LMNA gene [72]. These observations offer insights into the pivotal role of LAP2α in the regulation of chromatin organization. Mechanistically, our study demonstrates that LAP2α can interact with histone deacetylases to maintain a heterochromatic state at telomeres. These findings provide new insights into the role of nuclear architecture in regulating chromatin organization by LAP2α, particularly in its role of suppressing homology-directed repair in ALT cells or telomerase-positive cells in response to telomeric damage. Interestingly, the depletion of LAP2α is not sufficient to induce the formation of ALT features, indicating that it does not play a role in the initiation of ALT. Instead, it appears to balance homologous recombination to maintain telomere length after ALT activation.

Something deserves attention: a recent study reported that LAP2α could associate with Replication Protein A (RPA) and facilitate its deposition to damaged chromatin during homologous recombination [35]; however, our findings reveal a distinctive occurrence at telomere, that the disruption of LAP2α increases the recruitment of RPA to telomeric DNA. Previous research in mammalian cells has established that ALT is a break-induced replication (BIR)-related process, representing a late DNA synthesis persisting through the G2-M phase into mitosis, referred to as mitotic DNA synthesis (MiDAS) [73–75] However, classical homologous recombination (HR), primarily activated during the S and G2 phases, repairs genomic DNA double-strand breaks (DSBs) [76]. Our current data suggest that LAP2α‘s involvement in telomere homology-directed replication may differ from its role in ordinary genomic homologous recombination.

Methotrexate (MTX), a widely used drug in osteosarcoma treatment, has been identified as a potential molecule with the ability to increase heterochromatinization [57]. In our cell-based research, MTX induced enhanced heterochromatinization in the telomeric region, counteracting the chromatin decondensation caused by LAP2α depletion and subsequently suppressing ALT production. Furthermore, in the subsequent patient-derived xenograft (PDX) model, MTX effectively inhibited the growth of ALT-positive osteosarcoma tumors. However, the clinical subtyping of tumors based on ALT and the implementation of targeted therapies have not been actively pursued. Therefore, the use of MTX in the clinical treatment of ALT-positive osteosarcoma may potentially expand the benefits for relevant patients.

In conclusion, the findings from this study illuminate LAP2α‘s pivotal role in suppressing telomere-telomere recombination and Alternative Lengthening of Telomeres (ALT) activity. The study underscores LAP2α‘s significance in ALT formation, emphasizing the intricate interplay between telomere-nuclear envelope association and ALT activity, closely linked with heterochromatin structure. Notably, in a patient-derived xenograft (PDX) model, methotrexate (MTX) exhibited a specific inhibitory effect on LAP2α-low-expressing ALT-positive osteosarcoma tumors. This discovery opens up a promising avenue for targeted clinical applications, suggesting MTX as a potential therapeutic agent for the specific treatment of ALT-positive osteosarcomas. To conclude, the comprehensive nature of these findings not only emphasizes LAP2α‘s critical role in ALT but also positions MTX as a potential therapeutic option, potentially extending its applicability to other ALT-positive tumors. Certainly, further research and clinical investigations are warranted to explore the full scope of LAP2α and MTX as key players in the targeted therapy landscape for ALT-positive cancers.

## Materials and methods

### Cell culture and treatment

U2OS, HeLa, 293T, and SAOS2 cells were obtained from the American Type Culture Collection (Manassas, VA). Cells were cultured at 37 °C and 5% CO_2_. U2OS and 293T cells were grown in DMEM (Corning) supplemented with 10% fetal bovine serum (FBS, Gibco) and 1% penicillin/streptomycin (Hyclone). HeLa cells were grown in 1640 (Corning) with 10% FBS. SAOS2 cells were grown in McCoy’s 5A (Hyclone) with 15% FBS and 1% penicillin/streptomycin (Hyclone). All cell lines were identified by standardized short tandem repeat analysis. Mycoplasma was regularly examined during cell culturing, and no contamination occurred during this study. Sequences of the various siRNAs used in the study are: NC (negative control): 5′-UUCUCCGAACGUGUCACGUdTdT-3′; siLAP2α-1:5′-GCAACACAGAUAUUAU CAGdTdT-3′; siLAP2α-2:5′-GUCUAGAAGUGGCUAAGCAdTdT-3′.

### RT-qPCR

Total RNA was extracted with Eastep® Super Total RNA Extraction kit (Promega), and cDNA was prepared with HiScript II Q Select RT SuperMix for qPCR (Vazyme) following the manufacturer’s instructions. qPCR reactions were performed with ChamQ SYBR qPCR Master Mix (Vazyme, China). GAPDH was used for normalization. qPCR primers are listed in the Supplementary Table.

### Antibodies and western blot analysis

The antibodies used for western blot analysis were as follows: anti-GAPDH (affinity, AF0911, dilution 1:5000), anti-LAP2α (Abcam, catalog no. ab5162, dilution 1:1000). For immunoblotting, whole cell lysates were isolated using RIPA buffer, quantified using the Pierce TM BCA protein assay kit (Thermo Scientific, Waltham, MA), separated by SDS-PAGE and transferred onto nitrocellulose membranes. The lysate was resolved on SDS-PAGE gel. The separated proteins were then blotted on a nitrocellulose plus membrane. Membranes were blocked for 1 h in 5% non-fat dry milk in TBS/0.1% Tween-20 and incubated with appropriate primary antibody in blocking solution overnight at 4 °C. The membranes were washed 3 × 15 min with TBS/0.1% Tween-20 and incubated with appropriate secondary antibody in blocking solution for 1 h at room temperature. Chemiluminescence detection was performed using an ECL (MILLIPORE).

### Plasmid construction and lentivirus production

The pLenti-TRE-LAP2α-CBH-Tet-On@3G vector was a gift from Dr. Lei Shi (Tianjin Medical University, China), and the lenti-CRISPRv2 consisting of Flag-Cas9 enzyme and sgRNA were gifts from Yong Zhao (Sun Yat-sen University, Guangzhou). Briefly, the guiding sequence GTTAGGGTTAGGGTTAGGGTTA (referred to as sgTel in the text) was used to induce DSBs in telomeres. The scrambled sequence TGCTCCGTGCATCTGGCATC (referred to as sgScr in the text) was used as a control. For the production of the lentivirus, briefly, HEK293T cells plated in 10 cm dishes were transfected with 10 μg of expressing vectors, 7.5 μg of pSPAX2, and 2.5 μg of pMD2G using polyethyleneimine (PEI) (Polysciences, USA). Lentivirus supernatants were collected at 48 h post-transfection and clarified by filtration through 0.45 μm syringe filters.

### Immunofluorescence -fluorescence in situ hybridization (IF-FISH)

Cells were grown on a coverslip, washed with PBS, and fixed in 4% paraformaldehyde for 5 min at room temperature, and then permeabilized in 0.5% Triton X-100 at room temperature for 30 min. The cells were washed thrice with PBS and blocked with 5% goat serum for 1 h at room temperature. The cells were first incubated with primary antibody (anti-PML, Santa Cruz; anti-RPA2, Abcam; anti-RAD52, Santa Cruz; anti-53BP1, Abcam) overnight at 4 °C and then with secondary antibody conjugated with Alex488 or Alex555 for 1 h at room temperature. The coverslip was washed with PBST and fixed in 4% paraformaldehyde for 10 min. Dehydrated in 70%, 95%, and 100%, denatured at 85 °C for 5 min, hybridized with Cy3-labeled or Alex488-labeled CCCTAA PNA probe (Panagene) for 2 h at 37 °C, washed and mounted with the 4′,6-diamidino-2-phenyl-indole (DAPI, D3571, Life Technologies, Carlsbad, CA). Fluorescence was detected and imaged using a fluorescence microscope (Nikon, Tokyo, Japan).

### Chromosome orientation fluorescence in suit hybridization (CO-FISH)

After 54 h of the last transfection with siRNA, U2OS cells were incubated with BrdU for 18 h; Colchicine (1 μg/ml) was added 5 h before harvest. Cells were trypsinized and resuspended in a hypotonic solution of 0.075 M KCl incubated at 37 °C for 30 min. The cells were fixed thrice with methanol: acetic acid (3:1) for 10 min each time. The cells were then spread onto slides, digested with pepsin (1 mg/ml) for 40 s, and exposed to UV (365 nm, UVP-CL1000) in the presence of Hoechst for 35 min. The cells were treated with Exo III (200 U for 30 min at 37 °C), hybridized with G-rich probe (red, Cy3-labeled) and C-rich (green, Alex488-labeled) in sequence, mounted with DAPI, and observed using a fluorescence microscope (Nikon, Tokyo, Japan).

### Telomere quantitative-FISH (Q-FISH)

Cells were treated with 1 μg/ml colchicine for 3 h to enrich cells at metaphase. Cells were harvested and q-FISH was performed as previously described [77]. Cy3-labeled or Alex488-labeled (CCCTAA)_3_ PNA probe was used. Images were taken using a fluorescence microscope (Nikon, Tokyo, Japan). The fluorescence intensity of the telomeres was analyzed by ImageJ and the TFL-TELO program.

### C-circle assay

The C-circle assay was performed as described previously with minor modification [78]. Briefly, genomic DNA was extracted with Tissue gDNA isolation kit (Biomiga), and a total of 30 ng genomic DNA was used for amplification with Φ29 DNA polymerase (NEB) at 30 °C for 8 h followed by 65 °C for 20 min. The products were blotted onto nitrocellulose filter membrane, UV cross-linked, and hybridized with a DIG-labeled probe (CCCTAA)4 to detect C-circle amplification products. Blots were washed, exposed to Tanon 5200 (Tanon Science and Technology Co., Ltd, Shanghai, China), and quantified using ImageJ.

### Chromatin immunoprecipitation (ChIP) and dot blot

ChIP assays were carried out following the protocol from the truChIP Chromatin shearing Kit (Covaris). Briefly, cells were treated with 1% formaldehyde for 5 min at room temperature to crosslink proteins to DNA. The cell was harvested and lysed. After lysis, the nuclei pellet was collected and resuspended in CHIP buffer, and the chromatin was then sheared by AFA Focused-ultrasonicator (Covaris, Woburn, USA). A total of 25 μl of sheared chromatin was taken to analyze the shearing efficiency, and the remaining chromatin was subjected to chromatin immunoprecipitation. An aliquot of each sample was set aside as input control, while the remaining portion was subjected to immunoprecipitation with anti-H3K9me3 (Abcam) overnight at 4 °C, with IgG (5 μl/IP) as negative control. The complex of co-precipitation was captured by ChIP-Grade Protein G Dynabeads, and chromatin was eluted from antibody/Protein G Beads and reversed crosslinks. Subsequently, DNA was purified and eluted with PCR purification kits (QIAGEN, Hilden, Germany) for Dot Blot.

For dot blot, the purified DNA was denatured in 0.2 M NaOH at 65 °C for 10 min, neutralized with 2× SSC, and the samples were added to the membrane (Bio-Rad) using a dot-blotting apparatus loaded onto slot-blot Hybond N+ (GE Healthcare) membrane, UV cross-linked to the membrane, and pre-hybridized with DIG Easy Hyb solution (Roche) for 40 min and hybridized with DIG-labeled telomeric G-probe (TTAGGG)4 overnight at 42 °C. The membrane was washed twice in 2X SSC/0.1% SDS solution for 10 min, then twice in 0.1X SSC/0.1% SDS for 10 min at 42 °C. The hybridization signal was detected using the DIG detection system (Roche) and quantitated using the ImageJ software. Calculate the amount of telomeric DNA immunoprecipitated relative to the signal of the corresponding inputs. The ChIP values are represented as a percentage of the total input telomeric DNA,

### Proximity ligation assay (PLA)

The protein interaction studies were performed with PLA. A Duolink® In Situ Detection Reagent (DUO92002, Sigma, MO, USA) was used according to the manufacturer’s instructions. Briefly, cells were cultured on sterile coverslips in 24-well plates and treated with siRNA. After being fixed with 4% PFA and permeabilized using 0.5% triton X-100, cells were blocked in Duolink II solution for 1 h. The slides were incubated with anti-TRF1 antibody (1:200) or anti-TRF2 antibody (1:200) and anti-LAP2α antibody (1:500) at 4 °C overnight, followed by incubation with Duolink PLA anti-Mouse PLUS and PLA anti-Rabbit PLUS proximity probes. After washing the slides three times, the ligation reaction was done for 30 min, and the amplification was run for 100 min at 37 °C. Then, the slides were visualized using a fluorescence microscope (Nikon, Tokyo, Japan).

### ATAT-Seq

ATAC-seq was performed essentially as previously described [79]. Briefly, U2OS cells were treated with siNC or 50 μM siLAP2α RNA for 72 h. Then, cells were harvested and sent to Beijing Novogene Co., Ltd. ATAC-Seq were mapped to the human genome (hg19 from UCSC genome browser) using the Bowtie2 software package (version 2.3.0) [80], followed by standard quality control and adapter removal. For telomere read analysis, the maximum number of times of consecutive appearance of telomere sequence (TTAGGG or the reverse complement CCCTAA) with the exact match was counted.

### Immunohistochemistry staining

Tissue sections were deparaffinized in xylene and microwaved in 10 mM sodium citrate buffer (pH 6.0) to unmask the epitopes. Endogenous peroxidase activity was blocked by incubating for 10 min with 3% hydrogen peroxide in methanol. Immunohistochemical staining for LAP2α (1:200) was performed by using the indirect avidin biotin-enhanced horseradish peroxidase method according to the manufacturer’s instructions (Vector Laboratories, Burlingame, CA). After developing with 3,3’-diaminobenzidine, all sections were counterstained with hematoxylin and observed by microscope (200× magnification). Quantitative analysis of immunohistochemical staining was performed using the Image-Pro Plus software (version 6.2) program (Media Cybernetics, Inc., Rockville, MD).

### Patient-derived xenograft (PDX) mouse model

Six- to eight-week-old female C57BL/6 mice (weighing 14–19 g) were bought from Beijing Vital River Laboratories (China). The mice were kept in a specific pathogen-free facility at 21 ± 2 °C with a 12-h dark/light cycle. The mice were allowed to acclimate to these conditions for at least 7 days before starting experiments. All animal treatments were strictly in accordance with the International Ethics Guidelines and the National Institutes of Health Guidelines Concerning the Care and Use of Laboratory Animals. PDX animal studies were performed following guidelines approved by the Tianjin Medical University Institutional Animal Care and Use Committee (Tianjin, China). Human ALT+ and ALT− osteosarcoma tumor fragments were obtained from Tianjin Hospital and approved by the Medical Ethics Committee of Tianjin Hospital. The tumor fragments were cut into smaller fragments (2–3 mm) and then implanted into severe combined immune deficient (SCID) mice. A total of 48 mice were randomly assigned to four groups (12 mice per group): OS(ALT−) model, OS(ALT−)+MTX model, OS(ALT+), and OS(ALT+)+MTX model. MTX (0.5 mg/kg) was dissolved in PBS with 2.5% dimethyl sulfoxide (DMSO), 5% polyethylene glycol 400 (PGE 400) and 5% tween 80. MTX was administered to mice by oral gavage. To ensure adequate power to detect a prespecified effect, the sample size was chosen using the Power and Sample Size Program. Tumor volume measurements were obtained every 2 days, and tumor volume was calculated from measurements of 3 diameters of the individual tumor base using the ellipsoid formula: tumor volume (mm^3^) = (length × width × height × 0.52). Mice were monitored until tumor volume reached 1 cm^3^, at which time mice were euthanized and tumors were extracted. There were no animals excluded from analyses, and no blinding was carried out, but the data analysts were blinded to the groupings.

### Statistical analysis

Values from biological triplicate or more experiments are represented as mean ± SEM. The in vitro experiments were analyzed by unpaired Student’s *t*-tests using GraphPad Prism 8 to compare two groups of means and generate *p*-values. The standard designation of *p*-values was used throughout the figures (ns, not significant or *p* ≥ 0.05; *p < 0.05; **p < 0.01; ***p < 0.001; ****p < 0.0001). The data between the two compared groups meet the normal distribution and has a similar variance. The Kaplan–Meier method was used for survival analysis, and the differences in survival were measured using the log-rank test. Associations between the expression of LAP2α or ALT and OS survival were also evaluated with the Cox proportional hazards regression model at both univariate and multivariate levels.

## Supplementary information

Supplementary Figure and table

Western blot

## Data Availability

All data generated or analyzed during this study are included in this published article (and its supplementary information files).
