# Supplementary material for: LAP2α orchestrates alternative lengthening of telomeres suppression through telomeric heterochromatin regulation with HDAC1: unveiling a potential therapeutic target
Source: Cell Death Dis. 2024 Oct 19;15(10):761. doi: 10.1038/s41419-024-07116-4 (PMC11490576; doi:10.1038/s41419-024-07116-4)
Supplement: Supplementary file 1 — Supplementary Figure and table [file 41419_2024_7116_MOESM1_ESM.pdf]

Supplementary information

Supplementary Fig. S1. LAP2α interacted with shelterin complex in SAOS2 cells. (A) PLA of LAP2α and TRF1 or TRF2 in SAOS2 cells transfected with control or LAP2α siRNA. Red dots represent PLA signals. (B)Metaphase chromosome spreads and telomere FISH in MG32 cells. Representative images showing fragile sites (yellow arrows) and signal-free ends (SFE, white arrows). (C) Quantification of the fragile site and SFEs of MG32 cells. Error bars represent the mean ± SEM of three independent experiments. Two-tailed unpaired student's t-test was used to calculate P-values. ns, not significant.

Supplementary Fig. S2.Q-FISH analysis of relative telomere length in U2OS cells. (A) Representative images showing telomeres hybridized with Cy3-labelled telomere probe (red). (B) Frequency distributions of relative telomere length telomere length are shown above each panel.

Supplementary Fig. S3. Telomeric DNA double strands break induced by CRISPR-CAS9 SgTel system（A）Dot blot of C-circle assay performed with genomic DNA from Hela cells and(top) MG32 cells(bottom). (B) APBs formation upon sustained telomeric DSBs induced by CRISPR/Cas9 in Hela cells. APBs in indicated cells were visualized by hybridization using antibody to PML (green, IF) and telomeric probe (red, FISH). (C) Quantification of the percentage of positive cells with APBs of panel (B) Error bars represent the mean ± SEM of three independent experiments. Two-tailed unpaired student's t-test was used to calculate P-values. ****$p < 0.0001$. (D) Hela became C-circle positive induced by CRISPR/Cas9 in Hela cells.

Supplementary Fig. S4. Colocalization of RAD51 with telomeres was analyzed by IF-FISH using telomeric G-rich probe (green) and antibodies to RAD51(red) in U2OS.

Supplementary Fig. S5. qRT-PCR analysis of TERRA levels in the indicated SAOS2

cells. Error bars represent the mean ± SEM of three independent experiments. Two-tailed unpaired student's t-test was used to calculate P-values. ns not significant or $p \geqslant 0.05$; *$p < 0.05$; **$p < 0.01$; ***$p < 0.001$.

Supplementary Fig. S6. Clinical parameters of 39 osteosarcoma patients.

Supplementary Fig. S7. (A) Telomere dysfunction-induced foci (TIFs) shown by the colocalization of the telomere signals(Green) and DNA damage marker 53BP1(Red). The white arrow refers to TIFs. (B) Analysis of TIFs in all four groups. (C) Immunohistochemical staining of p16 in tumor tissues.

Supplementary Table 1 Primers used for RT-PCR

Supplementary Fig. S1

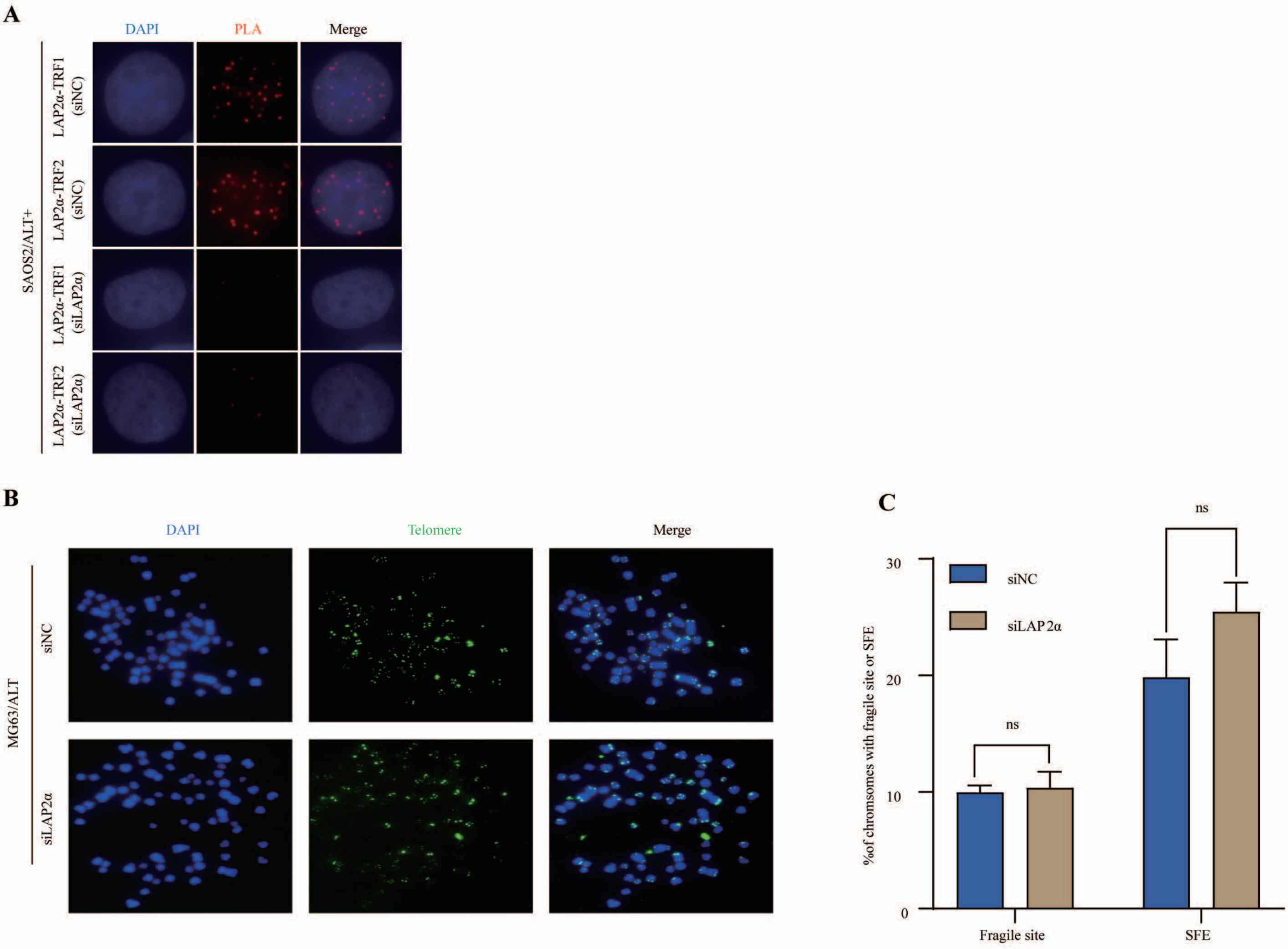

Supplementary Fig. S2

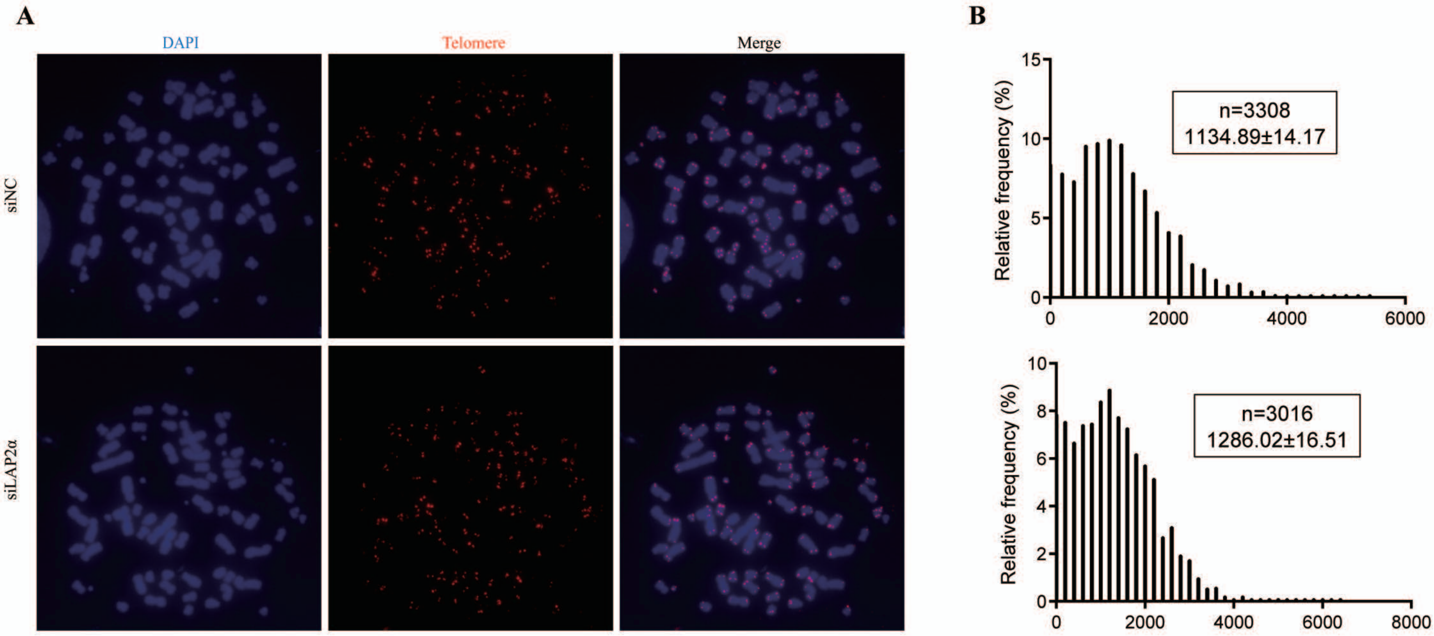

Supplementary Fig. S3

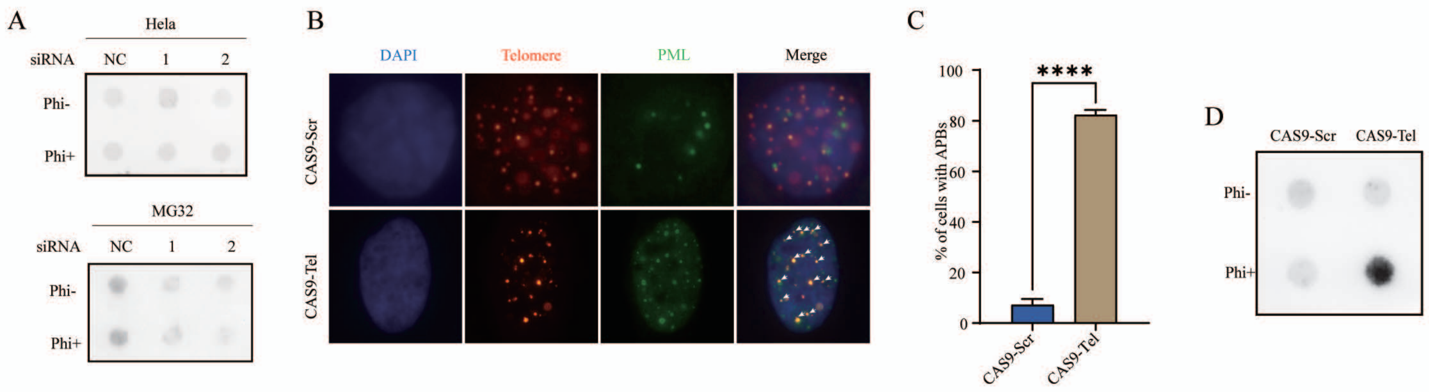

Supplementary Fig. S4

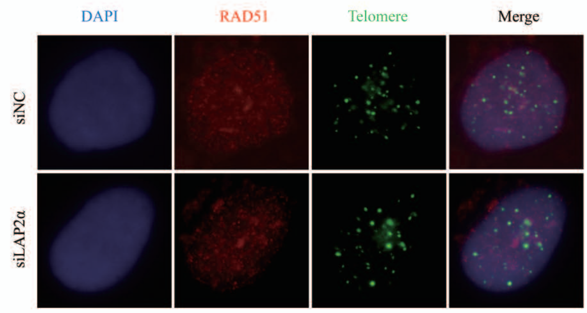

Supplementary Fig. S5

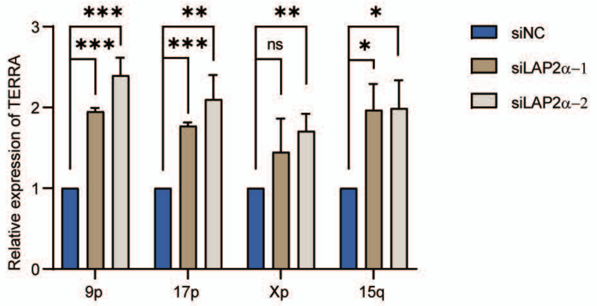

Supplementary Fig. S6

	ALT+		ALT-	
	Total	DSS Survived	Total	DSS Survived
Patients	21	12	18	14
Median age	34.5	35.8	33.6	37.9
Male	12	7	10	8
Female	9	5	8	6
Grade Ⅰ	15	11	11	10
Grade Ⅱ	4	1	5	3
Grade Ⅲ	2	0	2	1
Low	16	8	4	3
High	5	4	14	11

Supplementary Fig. S7

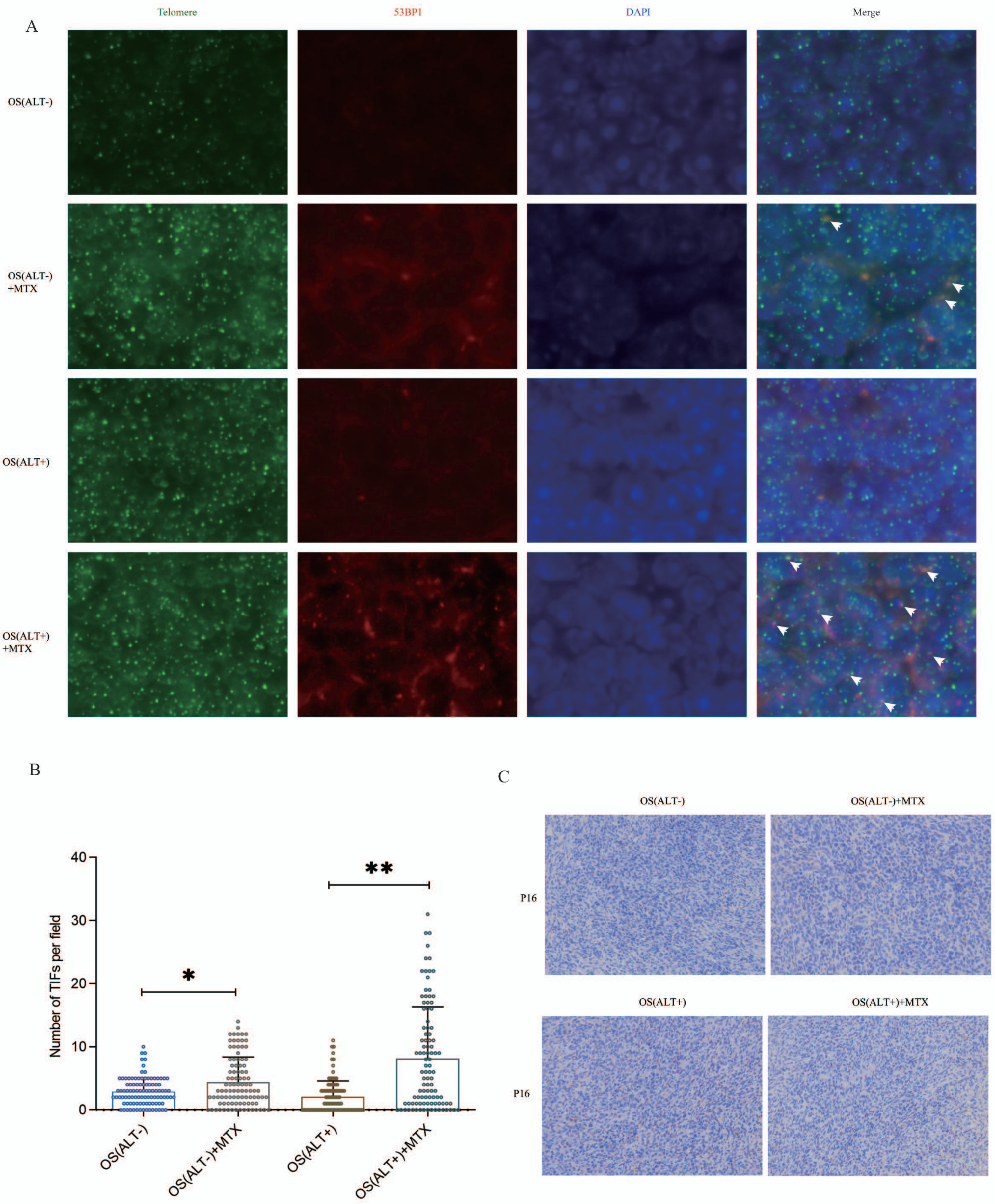

Supplementary Table 1

Primers used for RT-PCR

Region	Primer	Sequence (5'-3')
GAPDH	Forward	CGACAGTCAGCCGCATCTT
	Reverse	CCCCATGGTGTCTGAGCG
β-actin	RT-special	AGTCCGCCTAGAAGCATTTG
TERRA	RT-special	(CCCTAA)5
β-actin	Forward	TCCCTGGAGAAGAGCTACGA
	Reverse	AGCACTGTGTTGGCGTACAG
Chr 9p	Forward	GAGATTCTCCCAAGGCAAGG
	Reverse	ACATGAGGAATGTGGGTGTTAT
Chr 15q	Forward	CAGCGAGATTCTCCCAAGCTAAG
	Reverse	AACCCTAACCACATGAGCAACG
Chr 17p	Forward	CTTATCCACTTCTGTCCCAAGG
	Reverse	CCCAAAGTACACAAAGCAATCC
Chr Xp/Yp	Forward	GCAAAGAGTGAAAGAACGAAGCTT
	Reverse	CCCTCTGAAAGTGGACCAATCA
