# Supplementary material for: LAP2α orchestrates alternative lengthening of telomeres suppression through telomeric heterochromatin regulation with HDAC1: unveiling a potential therapeutic target
Source: Cell Death Dis. 2024 Oct 19;15(10):761. doi: 10.1038/s41419-024-07116-4 (PMC11490576; doi:10.1038/s41419-024-07116-4)
Supplement: Supplementary file 2 — Western blot [file 41419_2024_7116_MOESM2_ESM.docx]

Figure1C: U2OS（siLAP2α）Left blot: LAP2α Right：GAPDH





Figure1C: Hela（siLAP2α）Left blot: LAP2α Right：
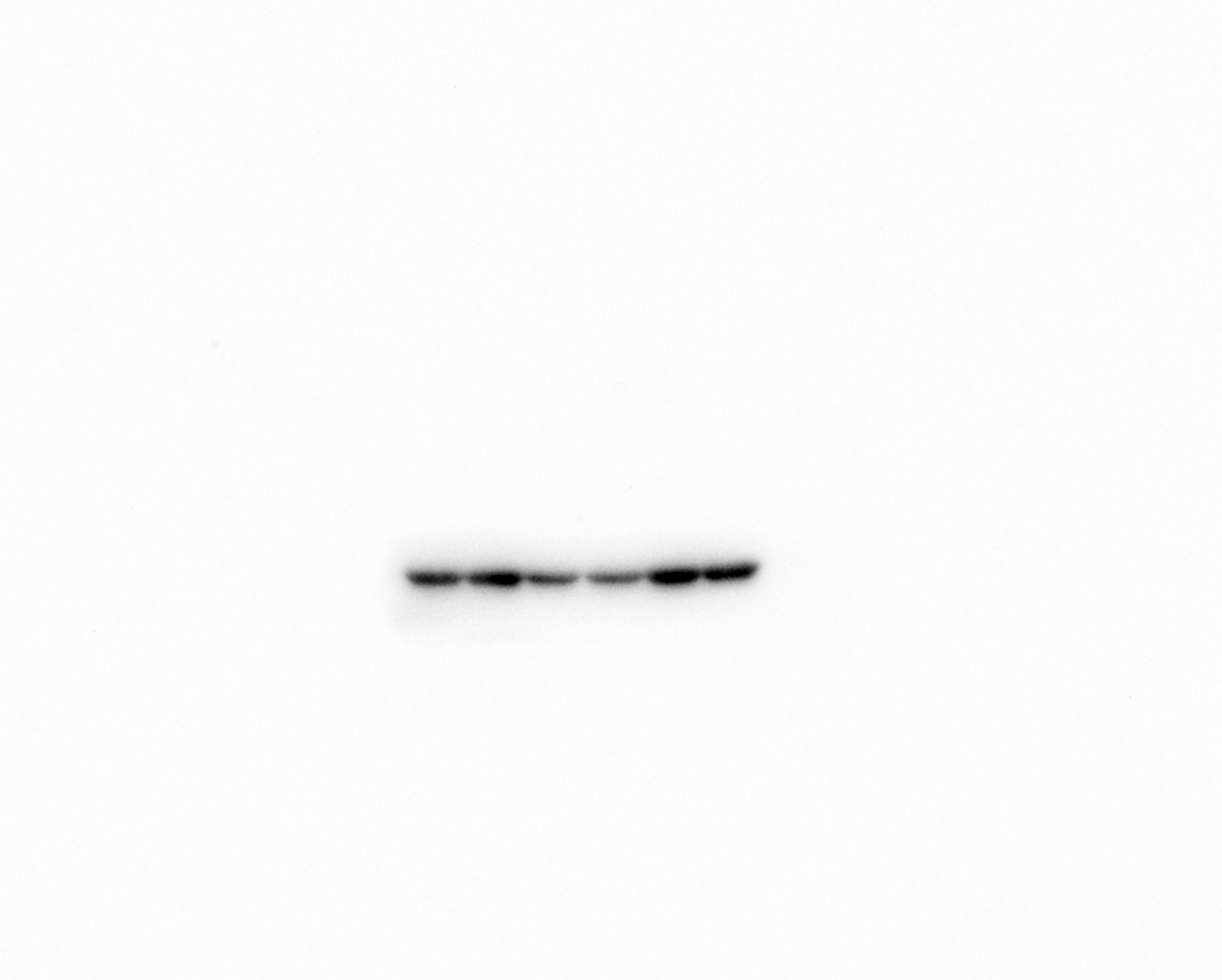

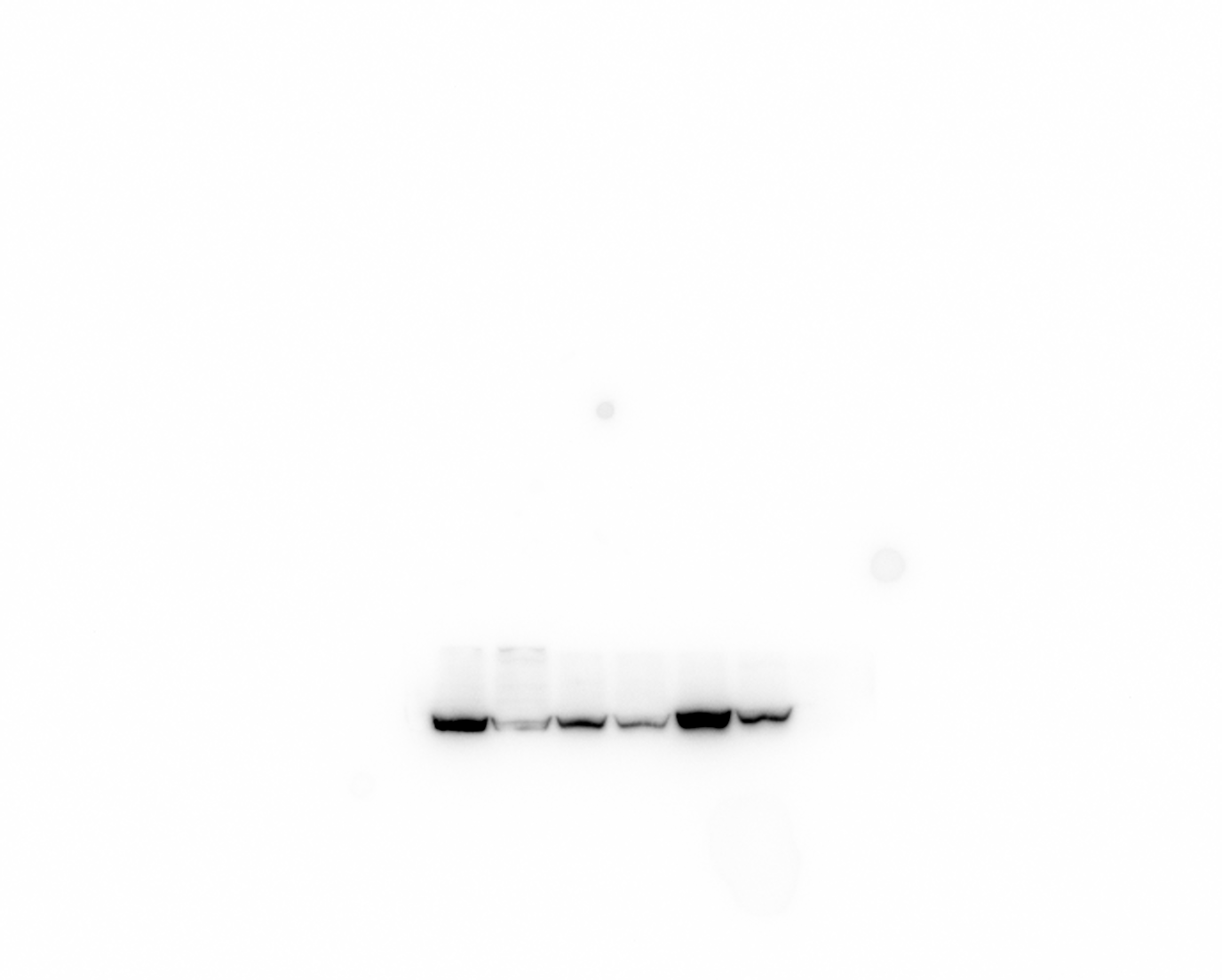
GAPDH

Figure2A: U2OS Left blot: LAP2α Right：GAPDH







Figure2A: SAOS2 Left blot: LAP2α Right：GAPDH




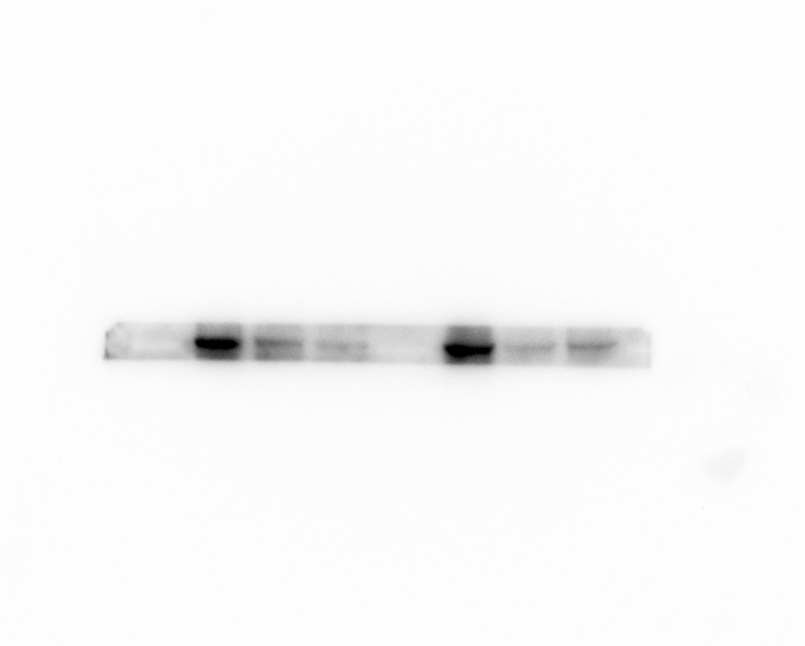


Figure3A: U2OS Left blot: LAP2α Right：GAPDH




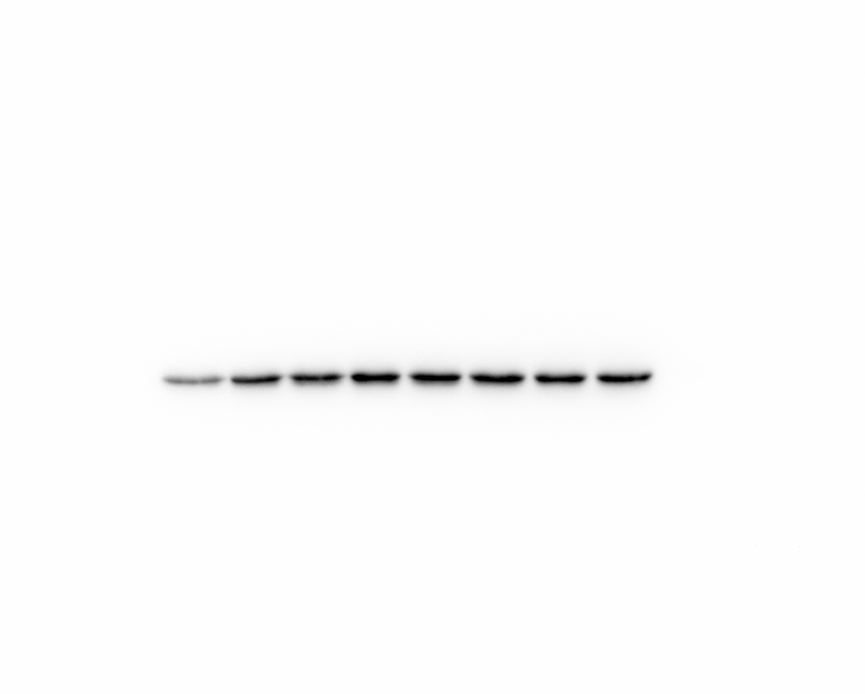
Figure3A:SAOS2 Left blot: LAP2α Right：GAPDH
